# Clinical Reporting of Magnetic Resonance Imaging, the Way Forward for Patients With Lumbar Disc Herniation: A Prospective Correlational Study

**DOI:** 10.7759/cureus.27232

**Published:** 2022-07-25

**Authors:** Ramavtar Saini, Anshu Sharma, Mirant B Dave

**Affiliations:** 1 Orthopaedics, Geetanjali Medical College and Hospital, Udaipur, IND

**Keywords:** spine examination, clinical reporting, mri spine, lumbar disc disease, lumbar disc degeneration

## Abstract

Background

Lower back pain (LBP) is a major cause of increasing years lived with disability. Many adults suffer from LBP once in their lifetime. Multiple environmental, genetic, and acquired factors lead to disc degeneration. Spinal stenosis can be caused due to bony, ligamentous, or discogenic origin. The majority of cases have a combined etiology of bony, ligamentous, and disc disease. Lumbar disc disease (LDD) has been mentioned by various terminologies in the literature. A standardized nomenclature is needed for better research and communication. Our study is based on the correlation between lumbar disc herniation (LDH) and magnetic resonance imaging (MRI) findings.

Methodology

A prospective observational study was undertaken on patients presenting with signs and symptoms suggestive of LDD to the Department of Orthopaedics at a tertiary care hospital in southern Rajasthan. The purposive sampling technique with a consecutive scheme was used. MRI is a valuable tool for diagnosing LDH.

Results

Many studies have concluded false-positive results for MRI in cases of LDH. Hence, interpretation with grading systems (Pfirrmann’s and Scizas grading) and correlation with clinical findings are mandatory for accurate diagnosis and management of patients.

Conclusions

We suggest clinicians adopt clinical reporting of MRI to improve the diagnostic accuracy with clinical and radiological correlation. Reporting can guide professionals in deciding the course of treatment in the form of conservative or surgical management.

## Introduction

Lower back pain (LBP) has been one of the leading contributors to years lived with disability (YLD) over the past few decades [1]. About 60% to 80% of adults suffer from LBP at least once in their lifetime [2,3].‎ Degeneration of intervertebral discs starts very early in life due to environmental factors and the normal aging process. Degeneration can lead to spinal stenosis, which can have a bony origin (spondylolisthesis, osteophytes, spondylosis, and facet hypertrophy), ligamentous origin (ligamentum flavum hypertrophy), or discogenic origin (lumbar disc disease, LDD). Most cases are a combination of bony, ligamentous, and disc diseases [4].

LDD is a common cause of pain in the lower back and radiculopathy in both the young and elderly [5,6]. Age-related, congenital, developmental, degenerative, traumatic, infective/inflammatory, neoplastic, or morphological variations are the major diagnostic categories of LDD [7].

Clinical presentation of patients with lumbar disc degeneration can include discogenic pain in the lower back, muscular weakness, sensory symptoms, or radicular pain. Radicular pain, muscular weakness, or sensory symptoms are usually distributed along the involved nerve roots. LBP is generally diffuse in the lumbar region or sometimes in the gluteal region or upper thighs [8].

The diagnosis of LDD has evolved from invasive myelography to non-invasive magnetic resonance imaging (MRI). The treatment options for LDD include conservative, selective nerve root blocks (SNRBs), endoscopic surgery, minimally invasive surgery (MISS), and open procedures. MRI is a reliable tool for diagnosing lumbar disc pathologies, classifying disc herniation, and planning treatment [9].

LBP has many clinical, economic, and social implications. The diametrically opposite treatment options, ranging from surgery to no surgery, make clinical decision-making necessary for good outcomes. Unfortunately, too many decisions to operate on the patient are made based solely on MRI.

Research on disc degeneration is complex because there is no standard definition for disc degeneration. Measures of disc degeneration available today lack precision and accuracy. It is impossible to measure the lifetime exposure of micromotion, vibration, or physical loading. A clear understanding of the cause of pain in disc pathology is yet to be explored.

Several studies have reported MRI to have high sensitivity for diagnosing lumbar disc herniation (LDH). However, a poor correlation of clinical findings with MRI findings in patients with LDH has been noted. MRI changes that appear to be LDH may be present in asymptomatic individuals. Hence, the correlation between imaging and clinical evaluation is essential for the accurate diagnosis and management of patients. With technological advances leading the medical profession, clinical reporting of MRI is necessary to avoid false diagnoses and select appropriate treatments.

Various terminologies are used in the literature to refer to LDD. A common nomenclature system must be standardized for better communication and research. This study is primarily based on the correlation between LDH and MRI findings.

## Materials and methods

A prospective observational study was undertaken on patients presenting with signs and symptoms suggestive of LDD to the Department of Orthopaedics at a tertiary care hospital in southern Rajasthan. The purposive sampling technique with a consecutive scheme was used. All patients with LBP who were with or without radiculopathy and were diagnosed with LDH on MRI were included in this study. The following patients were excluded: those with spinal trauma; those diagnosed with infective, inflammatory diseases, tumors, or cauda equina syndrome (CES); those with a history of spine surgery; and those with pacemakers and metal prosthesis in the body.

After an institutional ethical committee clearance (GU/HREC/EC/2019/1764), patients with clinical features of LBP with or without radiculopathy undergoing MRI evaluation were enrolled in the study. Demographic details of the patients were noted. Patients were thoroughly examined neurologically, and signs were recorded involving motor and sensory dermatomal levels. The clinical criteria to evaluate patients included the following: lower backache with radiation to the lower limb, radicular pain along a specific dermatome, a stretch test for nerve root tension (straight leg raise test, SLRT), presence of neurological deficit, and level of routine activity. All patients were evaluated using an MRI of the lumbar spine. Their clinical and radiological findings were noted. Blood tests (complete blood count (CBC), erythrocyte sedimentation rate (ESR), and C-reactive protein (CRP)) were done to rule out infective or inflammatory pathologies.

## Results

**Figure 1:**
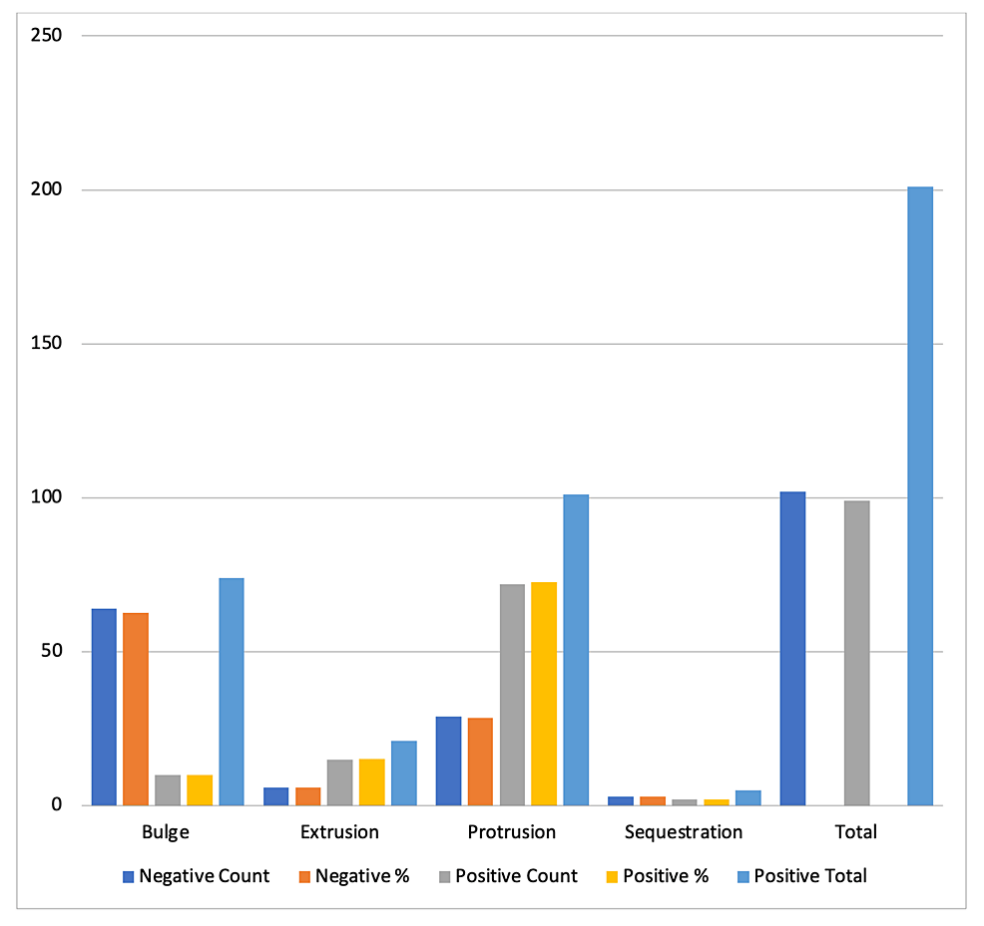
Lifestyle versus magnetic resonance imaging impression.

In our study, we found 41.4% of patients with a bulge, 8% with extrusion, 50.6% with a protrusion, and no patients with sequestration having acute LBP over fewer than three months. We also found 33.3% of patients with a bulge, 12.3% with extrusion, 50% with a protrusion, and 4.4% with sequestration having chronic LBP for more than three months (Figure 1; Table 1).

**Table 1:** Lifestyle versus magnetic resonance imaging impression.

Lifestyle	MRI disc disease	Central	Extraforaminal	Foraminal	Paracentral	Total
Active	Count	18	0	18	20	56
	%	17.30769	0	17.30769	19.23077	
Sedentary	Count	59	2	46	38	145
	%	40.68966	1.37931	31.72414	26.2069	
	Total	77	2	64	58	201
	X-squared = 2.8104, df = 3, p-value = 0.4218					

Our study showed that 49.25% of our patients had a positive SLRT result. Among the SLRT-positive group, 10.1% had a bulge, 72.7% had a protrusion, 15.1% had extrusion, and 2% had sequestration. Among the patients with a negative SLRT result, we found 62.7% with a bulge, 28.4% with a protrusion, 5.9% with extrusion, and 2.9% with sequestration on MRI.

**Figure 2:**
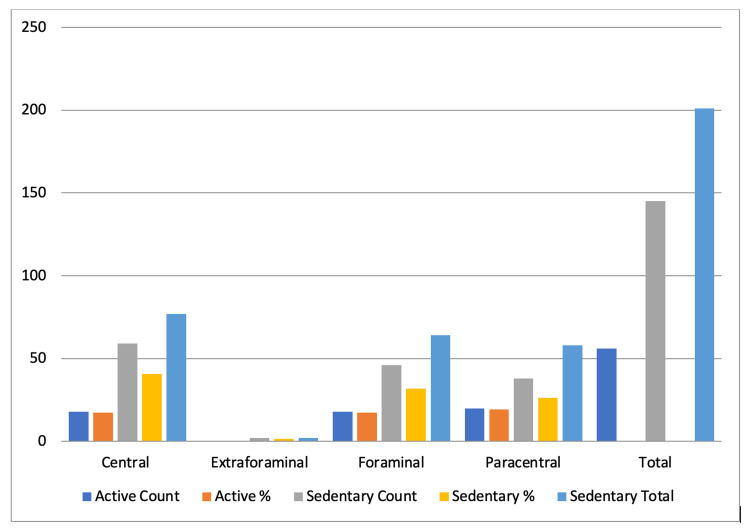
Straight leg raise test versus magnetic resonance imaging impression.

We classified each of our MRI findings according to Pfirrmann grading and found Grade III in 51.5% of our SLRT-positive patient group, Grade IV in 27.3%, Grade II in 16.2%, Grade V in 3%, and Grade I in 2%. Among the patient group with negative SLRT results, 37.3% were Grade II, 32.4% Grade III, 18.6% Grade IV, 8.8% Grade I, and 2.9% were Grade V (Figure 2; Table 2).

**Table 2:** Straight leg raise test versus magnetic resonance imaging impression.

SLR	MRI impression	Bulge	Extrusion	Protrusion	Sequestration	Total
Negative	Count	64	6	29	3	102
	%	62.7451	5.882353	28.43137	2.941176	
Positive	Count	10	15	72	2	99
	%	10.10101	15.15152	72.72727	2.020202	
	Total	74	21	101	5	201
	Chi-square = 61.74, df = 3, p-value = 2.499e-13					

We used Schizas grading to grade stenosis of the spinal canal. Among the SLRT-positive group, the A3 grade was found in 48.5% of patients, A2 in 19.2%, B in 19.2%, A4 in 11.1%, A1 in 2%, and C in no patients. In the SLRT-negative group, 39.2% were A2, 24.5% were A3, 12.7% were A1, 11.8% were B, 9.8% were A4, and 2% were C.

## Discussion

Definitions of disc pathologies have not been uniform. Various studies have used the following to define disc degeneration: autopsy, radiology, surgical samples, microscopy, and biochemical analysis. However, MRI is currently the most effective and accurate method for diagnosing LDD. In addition, quantitative measures such as disc space narrowing, bulging, desiccation, and signal intensity loss have been used for LDD. However, comparisons between studies are difficult due to a lack of uniformity.

Thompson et al. created the first grading scheme for disc degeneration. They used 15 cadaveric specimens to evaluate lumbar discs [10]. Battie et al. evaluated disc degeneration as bulges, protrusion, extrusion, disc narrowing, signal intensity changes, and high-intensity zones. Variable results have been reported in the literature due to a lack of uniformity in the definition of disc degeneration. Various studies have also noted differences based on the lumbar disc level. Schmorl’s nodes are more common at L1 to L3 levels, while degeneration is more common at L4 to S1 levels [11].

The nomenclature of LDD has been vague in the literature. Several deficiencies have been found in the terms defining lumbar spine conditions [12-14]. Spine scientists and practitioners across the world require a systematic and common nomenclature for the ease and precision of communication and data transfer [15].

In this study, we used the nomenclature system given by the American Society of Spine Radiology (ASSR), the North American Spine Society (NASS), and the American Society of Neuroradiology (ASNR). While multiple factors can be associated with LDD, the most studied in the literature include age, gender, environmental, and genetic factors.

Age is a factor intensely studied for LDD. Heine et al. showed degeneration in discs increasing from 0% to 72% linearly in patients aged 39 to 70 years [16]. Boos et al. showed degenerative scores increasing linearly between patients aged 2 to 88 years [17]. Gender differences in disc degeneration have been debated.

Multiple studies suggest a preponderance in males, while few studies suggest that females are more affected [18]. Some biochemical markers, such as calcium homeostasis factors and insulin growth factor binding protein 1, have been associated with lower disc space loss in females [19]. In our study, MRI impressions of LDH showed an almost equal distribution between males and females. A slight female preponderance was noted, which was insignificant. Our study had 104 females and 97 males. We found a slight female preponderance of Pfirrmann Grades III and V, while more male patients had Pfirrmann Grades I, II, and IV.

Our study found slightly more female patients with a positive SLRT result, which was insignificant. We also found male patients more commonly had a bilateral positive SLRT result.

In our patients, stenosis grading was done using Schizas grading, and we found an insignificant difference in the gender-based distribution. Environmental factors such as physical weightlifting, sports, and smoking have been correlated with LDD. Contrasting studies can be found for the dose-response relationship. In our study, we found a sedentary lifestyle to be a factor more commonly associated with LDD. For females, having a sedentary lifestyle was a major factor related to LDD. We found central, paracentral, and foraminal disc herniation more commonly associated with a sedentary lifestyle [20].

Many gene loci have been found to be associated with LDD. Sambrook et al. studied monozygotic twins for LDD and genetic predisposition. Degeneration summary scores of up to 74% heritability were found. Some studies found that a positive family history of LDD was a factor related to patients undergoing surgery for LDD.

The SLRT has been used for diagnosing discogenic pain since its invention in the 19th century. Our results showed that 33.1% of patients with radicular pain had a negative SLRT result, while 66.9% of patients with radicular pain had a positive SLRT result.

Radiculopathy is considered a clinical symptom of discogenic pain. In our study, out of the 53 patients without radiculopathy, 79.2% of patients had a bulge, 18.9% had a protrusion, 1.9% had extrusion, and no patients with sequestration did not have radicular symptoms. In 148 patients with radiculopathy, 61.5% had a protrusion, 21.6% had a bulge, 13.5% had extrusion, and 3.4% had sequestration. Our study had no patients with sequestration presenting without radicular pain. The majority of our patients had radiculopathy highly correlated with MRI impressions. However, we found that 26.4% of patients with MRI disc changes had no radicular pain. Out of the patients with no radiculopathy, most of the patients had Schizas grade A2; Schizas grade A3 was found in a majority of the patients with radiculopathy.

Sensory disturbances in patients with LDH are not well documented. Our study considered vibration sense, pinprick, fine touch, crude touch, temperature sense, and joint position for the diagnosis of sensory disturbances. We found that 13.9% of our patients had sensory disturbances. Moreover, we found that 42.8% of patients with sensory disturbances had central disc herniation, while the rest of the patients had paracentral and foraminal disc herniations. Sensory disturbances were absent in a majority of our patients.

The SLRT has been used for the clinical diagnosis of LDH for a long time. Pain originating from the hip, hamstrings, or sacroiliac joint can mimic radicular pain when performing SLRT. Performing a thorough clinical evaluation and proper history taking would reduce the inherent false-positive results of the SLRT. McCombe et al. reported that radiculopathy showed better agreement among clinicians when correlated with a dermatomal distribution [21]. The fact that “the patient’s response to the pain evoked by the SLR test is not uniform, and that muscle action potentials recorded during SLR and intentional resistance during the SLR test are different” [22] makes interpretation difficult in many patients.

Kostelijanets et al. found considerable interobserver variation among three observers in the measured angle at which pain was elicited on the SLRT [23]. Proper communication between the patient and clinician is mandatory for an accurate diagnosis.

The limitations of the sensitivity and specificity of the SLRT have been frequently discussed. The most sophisticated method may be using the “instrumental leg raising test to determine the extensibility and elasticity of the hamstring and the back muscles, and pelvic rotation in patients with LDH and in the normal population” [24] to determine the degree of movement in these tissues during the SLRT and to provide a better basis to interpret the test findings.

The hamstrings and medial hip rotation are limiting factors when interpreting SLRT results. Uncontrolled, medial hip rotation limits the use of the SLRT and increases tension and radiculopathy [25]. Bohannan et al. found pelvic rotation during the SLRT using cinematography that accompanies hamstring tightness [26]. Yamada and Yashizawa reported hamstring tightness more frequently in L5-S1 herniations, and gluteus maximus tightness more frequently with L4-L5 herniations.

Rebain et al. reported the SLRT to be highly specific and utilized it as a more reliable test for differential diagnosis. They also reported negative SLRT results may have a higher diagnostic value [27]. Vroomen et al. reported the sensitivity of the SLRT to be 85% and the specificity to be 52%. The authors suggested that SLRT sensitivity is overestimated, and its specificity is underestimated [28].

Our study suggested that 22.2% of our SLRT-positive patients had a central disc herniation, 34.3% had paracentral disc herniation, 42.4% had a foraminal disc herniation, and 1% had extraforaminal herniation. In contrast, our SLRT-negative patient group had 53.9% central, 23.5% paracentral, 21.6% foraminal, and 1% extraforaminal disc herniation.

LDH patients may have motor deficits. Hip flexion, knee extension, ankle dorsiflexion, the extension of the great toe, and ankle plantar flexion were tested for motor deficits. In our study, 20 patients had motor deficits, and 40% had extrusion, 35% had a protrusion, 20% had sequestration, and 5% had a bulge. Overall, 60% of these patients had a central disc disease, 30% had a paracentral disease, and 10% had a foraminal disease.

Interpretation of MRI and its grading is mandatory for an accurate diagnosis. Baker et al. found lumbar disc degeneration or herniation on at least one lumbar level in 35% of asymptomatic cases between patients aged 20 and 39 years, and all but one of the asymptomatic cases in patients aged 60-80 years old [29]. In our study, 48.6% of patients with a bulge had the Schizas grade of A2 as the associated finding in MRI. Moreover, 49.5% of patients with protrusion had the Schizas grade A3, while 61.9% of patients with extrusion had the Schizas grade B.

We did not consider patients with instability and facet arthropathy. MRI interpretation with findings other than disc pathologies is mandatory for an accurate diagnosis.

## Conclusions

LDD is a leading cause of YLD globally. Many researchers have reported lumbar disc-related disorders using different terminologies. A systematic nomenclature should be used by all researchers for uniformity in the literature. Recommendations for the lumbar disc nomenclature given by the combined task forces of the ASSR, the NASS, and the ASNR are the most accurate and widely used.

MRI is a valuable tool for diagnosing LDH. However, many studies have found false-positive impressions on MRI results for asymptomatic individuals. Hence, interpretation using grading systems (Pfirrmann and Schizas grading) and correlation with clinical findings are mandatory for the accurate diagnosis and management of patients. We suggest that clinical reporting of MRI be adopted by clinicians to improve the diagnostic accuracy using clinical and radiological correlations. Such reporting can guide medical professionals in deciding the course of management for conservative or surgical treatment.
